# The association of caffeine intake and prevalence of obesity among children and adolescents: A cross-sectional survey from NHANES 2011–2020 March

**DOI:** 10.1371/journal.pone.0300566

**Published:** 2024-06-03

**Authors:** Zi Rui Liu, Kai Cui

**Affiliations:** 1 Department of Early Childhood Education, Faculty of Early Childhood Education, Shaanxi Vocational and Technical College, Xi’An, China; 2 Department of Early Childhood Education, Faculty of Human Development, University Pendidikan Sultan Idris, Tanjong Malim, Perak, Malaysia; 3 Children’s Health Care, PLA 77th Group Hospital, Leshan, Sichuan, China; 4 Children’s Health Care, Ya Ba Ba Clinic, Xi’an, Shaanxi, China; GSVM Medical College, INDIA

## Abstract

**Background:**

Many studies have demonstrated the beneficial health effects of caffeine. However, its association with obesity prevalence and caffeine intake remains controversial. Notably, the impact of caffeine on children and adolescents needs to be more adequately represented in large-scale epidemiological investigations.

**Objective:**

This study examines the association between caffeine intake and obesity prevalence in children and adolescents aged 2 to 19.

**Methods:**

This study used the database from the National Health and Nutrition Examination Survey (NHANES, 2011–2020 March) to perform a cross-sectional study. A total of 10,001 classified children and adolescents were included in this analysis. All data were survey-weighted, and corresponding logistic regression models were performed to examine the associations between caffeine intake and the prevalence of obesity.

**Results:**

In a fully adjusted model, a per-quartile increase in caffeine intake was associated with a 0.05% increased prevalence of obesity. In the subgroup analysis, the multivariate-adjusted ORs (95% CIs) of the prevalence of obesity for per-quartile 1.3497 (1.2014, 1.5163) increments in caffeine intake were 1.5961 (1.3127, 1.9406) for boys and 1.4418 (1.1861, 1.7525) for girls, 1.5807 (1.3131, 1.9027) for white race and 1.3181 (1.0613, 1.6370), 1.0500 (0.6676, 1.6515) for the age of 2–5, 1.4996 (1.1997, 1.8745) for the age of 6–12, and 1.2321 (0.9924, 1597) for the age of 13–19.

**Conclusion:**

The study suggested that higher caffeine intake may have a protective effect against obesity in specific subgroups, particularly among no overweight individuals. However, the association was not significant in other groups, indicating the need for a nuanced understanding of caffeine’s impact on obesity in diverse populations.

## Background

The escalating prevalence of obesity among children and adolescents globally presents a serious public health concern [1]. Recent reports indicate a dramatic increase in obesity rates among this demographic, escalating from 11 million to 124 million in just four decades [2], with significant implications for both psychological well-being and the prevalence of chronic diseases such as diabetes, cardiovascular diseases, and certain cancers [3, 4]. Therefore, this alarming trend underscores the urgency to identify and understand the factors associated with obesity during childhood and adolescence.

Amidst various dietary components, caffeine stands out due to its ubiquitous in commonly consumed beverages, such as chocolate milk, energy drinks, hot chocolate, tea leaves, and colas [5]. Humans have a long history of using caffeine, and currently, nearly 73% of children and adolescents in the U.S. consume caffeine every day [6]. Notably, a vast majority of children and adolescents in the U.S. are regular caffeine consumers. This widespread consumption, particularly of caffeine-rich drinks, has recently surged, raising questions about its health impacts [7].

Caffeine, a stimulant frequently encountered in dietary sources, exerts pronounced effects on the central nervous system, enhancing alertness and mitigating fatigue. Its presence extends into the realm of pharmacology, where it is incorporated into treatments for conditions like migraines, due to its vasoconstrictive properties, and utilized for its diuretic effects [8–10]. The diverse applications of caffeine, from a common dietary component to a therapeutic agent, reflect its broad impact on human health. Our study delves into the dietary dimension of caffeine, specifically its correlation with obesity rates among the youth, while acknowledging the broader spectrum of its physiological implications.

The role of caffeine in modulating body weight, especially among children and adolescents, has elicited mixed findings from the scientific community [11, 12]. Some evidence suggests that caffeine may play a protective role against weight gain, yet the current literature lacks a consensus, particularly regarding this age group [13, 14]. Addressing this ambiguity, our research harnesses the extensive data of NHANES from 2011 to March 2020. Through this analysis, we aim to dissect the potential associations between caffeine consumption and obesity prevalence, thereby contributing to a deeper understanding of dietary influences on pediatric health.

## Methods

### Study population

**Fig 1:**
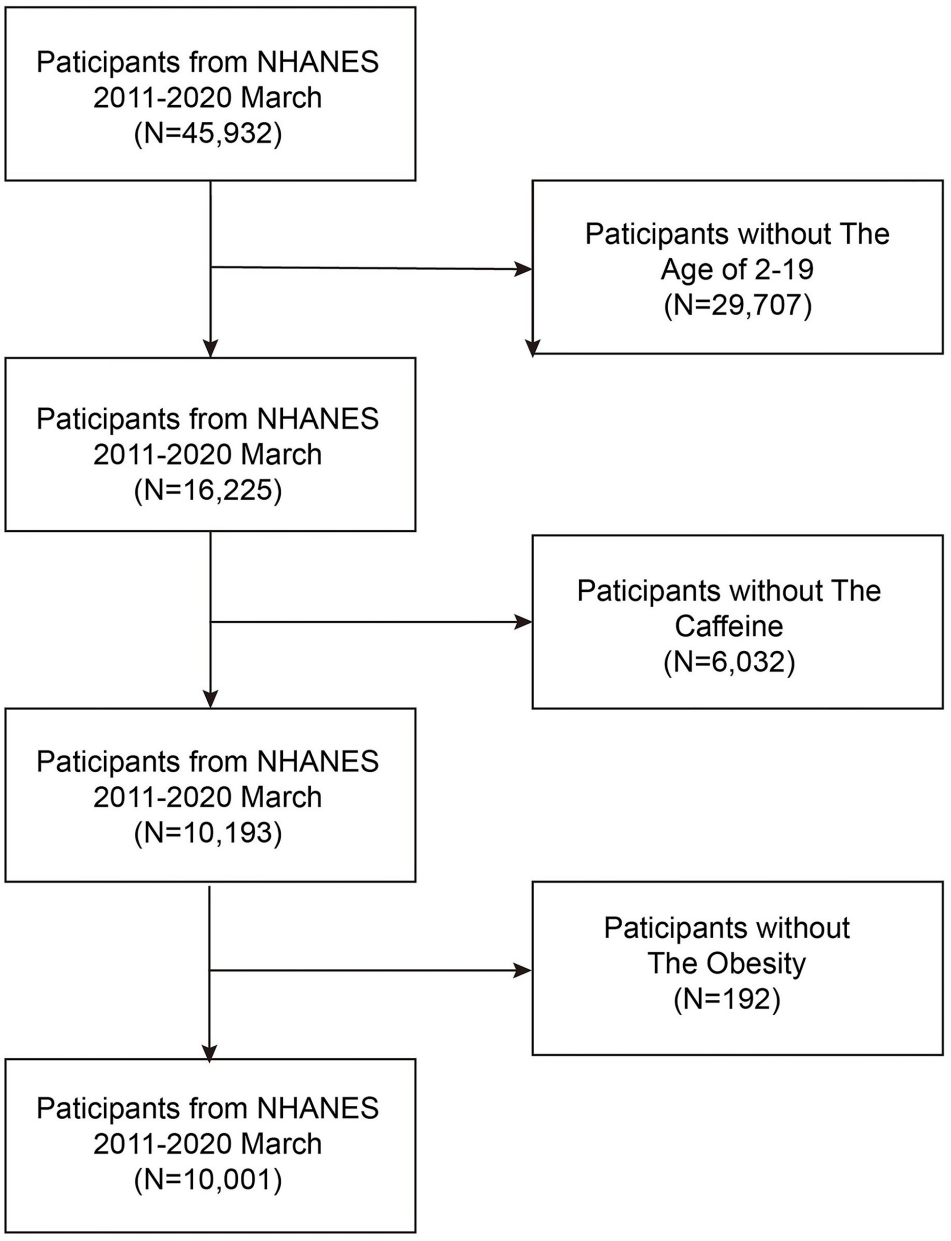
The flowchart of participants.

All the databases could be obtained from the NHANES website (https://wwwn.cdc.gov/nchs/nhanes/Default.aspx), which is a nationally representative survey of nutrition and health statistics in the United States. The National Center for Health Statistics (NCHS) Ethics Review Board approved the whole program, and all participants signed informed consent. The secondary hand analysis did not require additional Institutional Review Board approval. This study analyzed the data from 2011–2020 March. This study had 16,225 participants between the ages of 2 and 19 years. Then, the participants who had no data on caffeine and body mass index (BMIC) were also excluded. The final study population of this study was 10001. The flowchart of the participants is shown in Fig 1.

### Exposure variable

All NHANES participants were eligible for two 24-hour dietary recall interviews. The first dietary recall interview was conducted in person at the Mobile Examination Center (MEC), and the second interview was conducted by telephone 3–10 days later. Daily totals of nutrients/food components for all foods were calculated for NHANES data collection using the USDA Dietary Study Food and Nutrition Database, including approximately 50 coffee beverages, 30 teas, and caffeinated beverages. Therefore, the analysis used the average caffeine intake from two total nutrient intake recalls. The main result of the study was the answer to the question ‘The relationship between caffeine and obesity among children and adolescents.

### Outcome variable

NHANES measures data are used to monitor trends in infant and child growth to estimate the prevalence of overweight and obesity in the U.S. children, adolescents, and adults and to examine the associations between body weight and the health and nutritional status of the U.S. population. BMI was calculated as body weight in kilograms divided by height in meters squared. The BMI of children and adolescents is based on age and gender and is often called the age-based BMI (BMI-for-age). The Centers for Disease Control (CDC) compiles these BMI values into age-based BMI growth charts for boys and girls to obtain a percentile ranking. BMI-for-age illustrates the weight status categories used for children and adolescents (including underweight, normal healthy weight, prevalence for overweight, and overweight categories) [13]. This study used the data from the NHANES 2011–2020 March ‘Body Measures–BMI Category—Children/Youth’ for children and adolescents as the research sample.

### Covariables

Based on a previous study on caffeine intake and obesity prevalence [14], the following variables were included as covariates: body mass index-children and adolescents (BMIC), energy, protein, total sugar, total fat, cholesterol, vitamin B6, vitamin C, calcium, phosphate, zinc, potassium, magnesium, sodium, selenium, and caffeine. The mean intake of the above dietary factors from the average of two total nutrient intakes was used in the present analysis. Socioeconomic characteristics, including age, sex, race, and the ratio of family income to poverty, were also associated with the prevalence of obesity, which was included in this analysis.

### Missing covariables

After excluding samples of 2–19 years old missing data about caffeine intake or obesity, addresses for 78.23% (35,931 out of 45,932) of the participants could not be geocoded and contributed to missing data in cross-sectional analyses. As such, 8 multiple imputations using fully conditional specifications addressed potential biases arising from item nonresponse.

### Statistical analysis

All statistical analyses were conducted according to CDC guidelines (https://wwwn.cdc.gov/nchs/nhanes/tutorials/default.aspx). Sample weight was considered and assigned to each participant [15]. Continuous variables are the mean [standard deviation (SD)]. Categorical variables are presented as frequencies or percentages. Weighted analysis of variance (ANOVA; for continuous variables) or the chi-square test (for categorical variables) was used to calculate the differences among different groups. The logistic regression analysis treated characteristics with three or more categories as indicator variables.

Weighted logistic regression was used to calculate odds ratios (ORs) and 95% confidence intervals (CIs) for overweight and obese individuals for each quartile of caffeine intake, and this study calculated three different logistic regression models. Model 1 was the crude model, and model 2 was adjusted for age (continuous), sex, and race. Model 3 included the covariates of model 2 with additional adjustments for the ratio of family income to poverty, energy, protein, total sugar, total fat, cholesterol, calcium, phosphate, sodium, potassium, magnesium, zinc, and vitamins B6, C, and D, and copper. Caffeine was adjusted for total energy intake with a residual model. Subgroup analysis was performed by gender, race, and ratio of poverty income. Weighted stratified line regression models were used to perform subgroup analysis, and interaction terms were used between subgroup indicators to test the effect modification in subgroups.

A value of p < 0.05 was considered significant. All statistical analyses were conducted in SAS version 9.4 (SAS Institute, Cary, North Carolina) or SAS-callable SUDAAN (RTI International, Raleigh, North Carolina) with combined dietary sample weights for nonresponse and the complex sampling design.

## Results

### Baseline characteristics of the participants

The population characteristics and other covariates of the weighted distribution of included participants by caffeine intake quartiles are shown in Table 1. We organized the study population into age groups that coincide with key educational stages—preschool (2–5 years), primary (6–12 years), and middle school (13–19 years)—to reflect their distinct dietary patterns and developmental milestones, which are integral to analyzing the impact of caffeine on obesity. 50.24% of them were boys, and 49.76% were girls. The weighted rate of obesity was 34.87%. The average caffeine intake of the overall participants was 20.43 ± 18.86 mg/day, and the averages of caffeine intake for quartiles 1–4 were 0.00 ± 0.03, 1.79 ± 0.95, 9.86 ± 4.90 and 70.09 ± 69.55, respectively.

**Table 1:** Baseline characteristics of the participants.

		Caffeine Intake				P value
	Overall	Quartile 1	Quartile 2	Quartile 3	Quartile 4	
		≤236.25	236.25–472.5	472.5–708.05	≥708.05	
Age						<0.001
2–5		793	788	590	161	
6–12		865	1139	1273	872	
13–19		732	570	749	1469	
Caffeine, mg/day [M(SD)]	20.43 (18.86)	0.00 (0.03)	1.79 (0.95)	9.86 (4.90)	70.09 (69.55)	<0.001
Rate of Obesity	34.87%	33.80%	31.60%	33.31%	40.77%	<0.001
Gender						0.020
Male	50.24%	49.71%	48.78%	49.58%	52.88%	
Female	49.76%	50.29%	51.22%	50.42%	47.12%	
Race						<0.001
Mexican American	507	434	466	598	530	
Other Hispanic	274.25	263	248	319	267	
Non-Hispanic White	674.5	450	638	720	890	
Non-Hispanic Black	650.5	826	717	557	502	
Other Race—Including Multi-Racial	394	417	214	418	313	
The ratio of Family Income to Poverty	1.99 (1.54)	1.88 (1.49)	2.10 (1.61)	2.04(1.57)	1.95 (1.48)	<0.001
Daily Intake [M (SD)]						
Energy (Kcal)	1832.67 (669.36)	1601.75 (636.45)	1741.35 (575.03)	1889.97 (675.76)	2097.60 (790.18)	<0.001
Protein (gm)	66.92 (29.19)	61.87 (29.35)	64.41 (25.50)	67.45 (27.85)	73.95 (34.06)	<0.001
Total Sugar (gm)	108.78 (48.99)	88.29 (44.38)	101.50 (41.48)	113.80 (48.51)	131.52 (61.59)	<0.001
Total Fat (gm)	69.31 (31.05)	59.50 (29.35)	65.21 (26.77)	72.22 (31.69)	80.29 (36.39)	<0.001
Cholesterol (mg)	224.00 (153.60)	207.19 (151.22)	211.28 (136.97)	223.90 (150.01)	253.61 (176.21)	<0.001
Vitamin B6 (mg)	1.71 (0.94)	1.62 (0.89)	1.66 (0.75)	1.69 (0.83)	1.85 (1.29)	<0.001
Vitamin C (mg)	76.67 (64.46)	83.71 (75.97)	79.86 (59.58)	76.72 (61.59)	66.40 (60.71)	<0.001
Calcium (mg)	969.21 (476.25)	881.53 (461.23)	987.19 (432.39)	1017.95 (491.61)	990.17 (519.77)	<0.001
Phosphorus (mg)	1218.67 (491.88)	1103.66 (481.80)	1195.86 (435.22)	1257.69 (498.08)	1317.45 (552.38)	<0.001
Magnesium (mg)	227.49 (93.05)	210.74 (94.93)	223.89 (81.20)	235.54 (94.01)	239.80 (102.05)	<0.001
Zinc (mg)	9.60 (4.79)	8.80 (4.99)	9.31 (4.18)	9.85 (4.57)	10.43 (5.40)	<0.001
Copper (mg)	0.90 (0.45)	0.80 (0.42)	0.88 (0.46)	0.96 (0.46)	0.97 (0.45)	<0.001
Sodium (mg)	2492.03 (1249.28)	2676.89 (1240.84)	2800.42 (1131.75)	2960.30 (1237.32)	3330.50 (1387.19)	<0.001
Potassium (mg)	2128.72 (841.44)	2002.22 (830.37)	2110.49 (755.58)	2179.46 (854.07)	2222.71 (925.74)	<0.001
Selenium (mg)	94.83 (44.13)	88.20 (43.85)	91.58 (41.30)	95.15 (41.72)	104.40 (49.66)	<0.001

Caffeine and dietary confounders (e.g., minerals and vitamins) were adjusted for total energy intake with a residual model.

M, mean values; SD, standard deviation.

Compared to Quartiles 1–3, participants in Quartile 4 had a higher rate of obesity (40.77%, p < 0.001). In addition, in terms of daily intake, participants in Quartile 4 had a higher intake of energy, protein, total sugar, total fat, cholesterol, vitamin B6, phosphate, magnesium, zinc, copper, sodium, potassium, and selenium and a lower intake of vitamin C and calcium.

### Association between caffeine intake and the prevalence of obesity

The association between caffeine intake and the prevalence of obesity is shown in Table 2. In model 1, a crude model, the prevalence of obesity negatively correlated with caffeine intake. The quartile analysis suggested that a per-quartile increment of caffeine intake was associated with a 0.03% decreased prevalence of obesity. In model 2, adjusted for gender, age, and race, the negative association between them exhibited a weakening trend.

**Table 2:** Association between caffeine intake and obesity.

Caffeine Intake	OR (95% CI), P value		
	Model 1	Model 2	Model 3
Quartile 1	1.0000 (reference)	1.0000 (reference)	1.0000 (reference)
≤236.25			
Quartile 2	1.0115 (0.9258, 1.1052) 0.7993, <0.0001	1.0135 (0.9250, 1.1105) 0.7737, <0.0001	1.0131 (0.9166, 1.1196) 0.7993, <0.0001
236.25–472.5			
Quartile 3	1.0171 (1.0005, 1.0340) 0.0440, <0.027	1.0088 (0.9918, 1.0262) 0.3114, <0.0001	1.0077 (0.9891, 1.0267) 0.4203 <0.0001
472.5–708.05			
Quartile 4	1.0008 (0.9996, 1.0019) 0.1765, <0.0001	1.0009 (0.9997, 1.0022) 0.1344, <0.0001	1.0020 (1.0005, 1.0036) 0.0086 <0.0001
≥708.05			
Per Quarter	1.0009 (0.9997, 1.0020) 0.1363, <0.0001	1.0011 (0.9999, 10023) 0.0709, <0.03	1.0022 (1.0009, 1.0036) 0.0015, <0.0001
P Trend	<0.0001	<0.0001	<0.0001

Caffeine and dietary confounders (e.g., minerals and vitamins) were adjusted for total energy intake with a residual model.

Model 1: crude model.

Model 2: adjusted for gender, age, and race.

Model 3: adjusted for gender, age, race, the ratio of family income to poverty, total intake (from dietary and supplements), energy, protein, total sugar, total fat, cholesterol, vitamin B6, vitamin C, calcium, phosphorus, zinc, copper, sodium, phosphorus, and selenium.

However, in model 3, the model adjusted for all relative covariates, including gender, age, race, the ratio of family income to poverty, total intake (from dietary and supplements), energy, protein, total sugar, total fat, cholesterol, vitamin B6, vitamin C, calcium, phosphorus, zinc, copper, sodium, phosphorus and selenium, and the prevalence of obesity appeared to be positively correlated with caffeine intake. In model 3, the OR (95% CI) of the prevalence of obesity for quartile 4 vs. quartile 1 was 1.0020 (1.0005, 1.0036), and per-quartile increments of caffeine intake were associated with a 0.05% increased prevalence of obesity.

**Fig 2:**
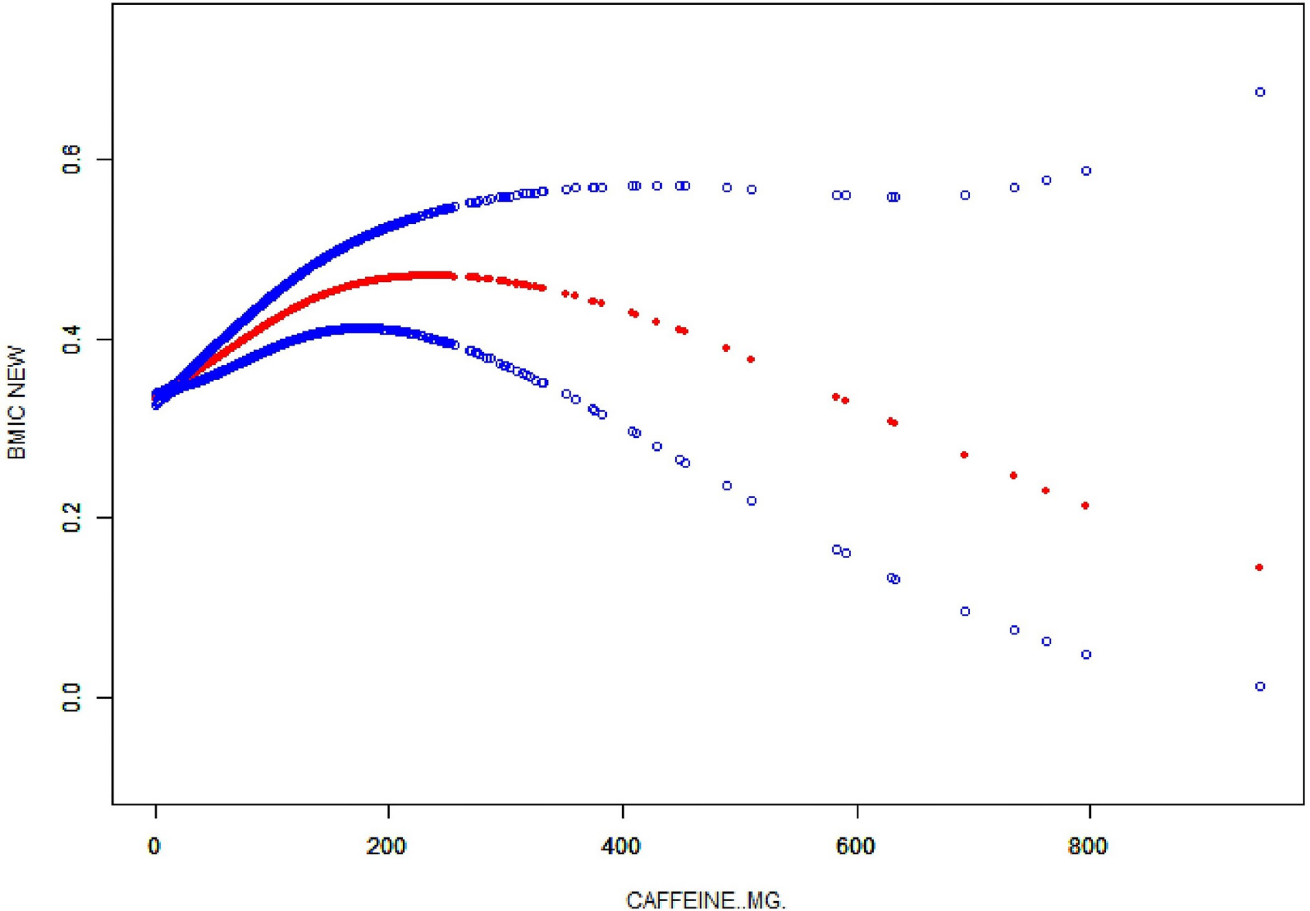
Association between caffeine intake and obesity.

**Fig 3:**
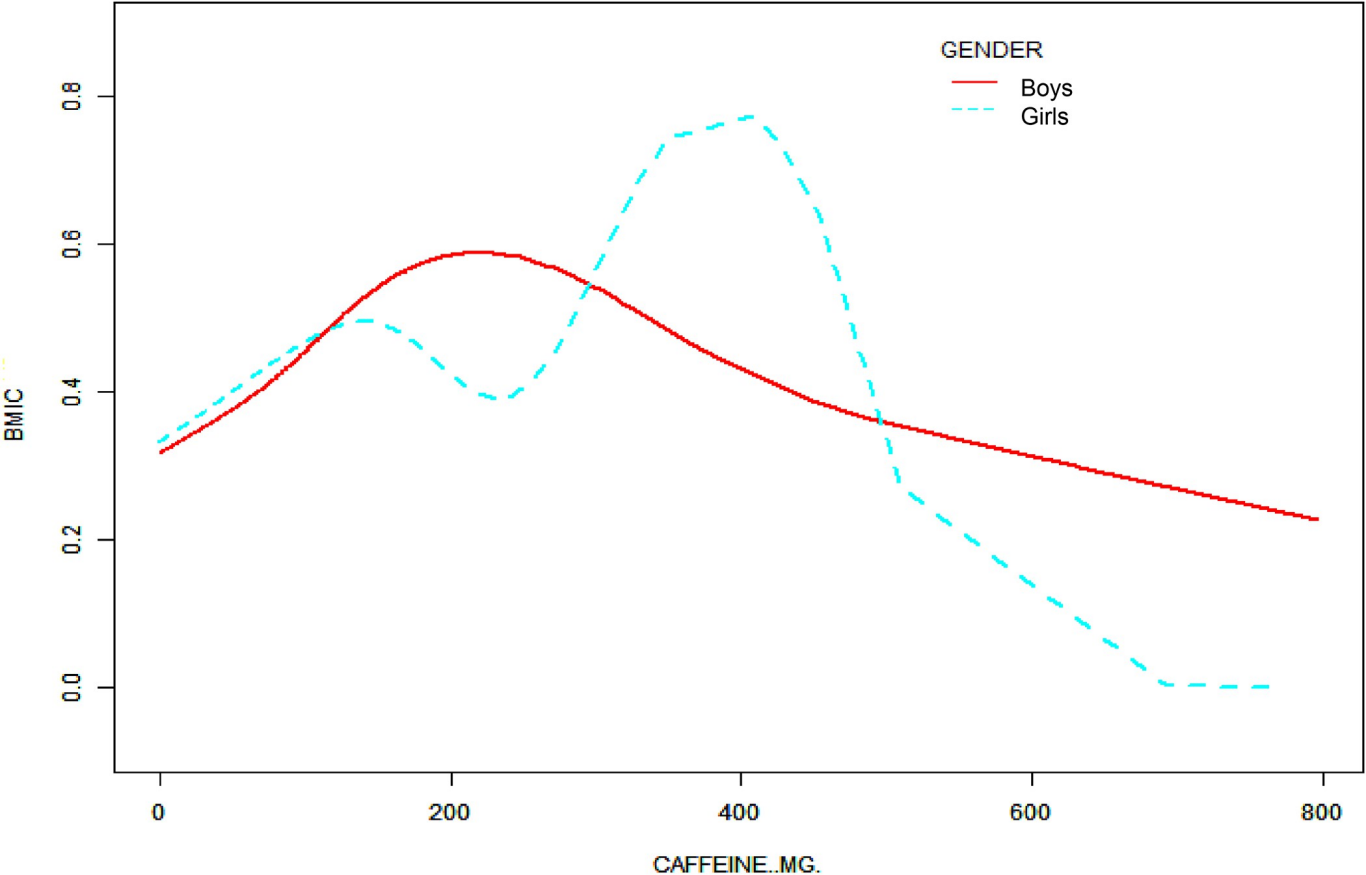
The association between caffeine intake and obesity stratified by sex.

Figs 2 and 3 illustrate the nonlinear association between caffeine intake and the prevalence of obesity, depicted through a curved line rather than a simple linear trajectory. In these figs, the initial rise in the prevalence of obesity with low caffeine intake is followed by a decline and subsequent with low caffeine intake is followed by a decline and subsequent increase, forming a U-shaped curve. This pattern suggests a dose-response relationship, where varying levels of caffeine intake exert differential effects on obesity prevalence. To construct these figs, we employed polynomial regression analysis, which allows for the modeling of non-linear relationships between the independent variable, caffeine intake, measured in milligrams, and the dependent variable, the body mass index (BMI) categorization of obesity. This analytical approach enabled the visualization of the potential threshold effect of caffeine on obesity risk, with data stratified by age groups in Fig 2 and by gender in Fig 3, thereby providing a more nuanced understanding of these dynamics.

A nonlinear relationship between caffeine intake and obesity was observed using a two-stage linear regression model with an inflection point of 479.15, as shown in Table 3. After stratifying the analysis by gender, age, race, the ratio of family income to poverty, total intake (from dietary and supplements), energy, protein, total sugar, total fat, cholesterol, vitamin B6, vitamin C, calcium, phosphorus, zinc, copper, sodium, phosphorus, and selenium, an inverted U-shaped curve was also present in boys and girls, with inflection points of 223.36 and 411.15, respectively.

**Table 3:** Threshold effect analysis of caffeine and obesity.

	Adjusted OR (95% CI), *p* Value
Obesity	
Inflection Point	26.99
Caffeine < 26.99	1.0022 (1.0009, 1.0013) **
Caffeine ≥ 26.99	0.9999 (0.9997, 1.0012)
Log likelihood radio	0.0008
Boys	
Inflection Point	223.36
Caffeine < 223.36	1.0037 (1.0021, 1.0054)
Caffeine≥223.36	1.0015 (0.9987, 1.0038)
Log likelihood radio	<0.0001
Girls	
Inflection Point	411.15
Caffeine <411.15	1.0025 (1.007, 1.0142) **
Caffeine≥411.15	0.9997 (0.9993, 10040)
Log likelihood radio	<0.0055

### Subgroup analyses

In our study, we performed a detailed subgroup analysis, adjusting caffeine intake for total energy intake using a residual model. This approach effectively controls for the potential confounding effect of overall energy consumption. We conducted stratified analysis by sex, race, and age to delve deeper into the factors influencing the association between caffeine intake and obesity prevalence in children and adolescents.

The results of these stratified analyses are concisely depicted in Fig 1, presents the subgroup analysis results of the crude model (model 1) and fully adjusted model (model 3). The detailed analysis results are shown in Table 4. In model 3, the fully adjusted model, the ORs (95% CIs) of the prevalence of obesity for quartile 4 vs. quartile 1 were 1.5961 (1.3127, 1.9406) for boys and 1.4418 (1.1861, 1.7525) for girls. A per-quartile increment of caffeine intake was associated with 20.14% and 32.13% increased prevalence of obesity for boys and girls, respectively.

**Table 4:** Subgroup analysis.

	**OR (95% CI), P value**												
**Caffeine Intake**	**Boy**							**Girl**					
	**Model 1**		**Model 2**			**Model 3**		**Model 1**		**Model 2**		**Model 3**	
Quartile 1	1.0		1.0			1.0		1.0		1.0		1.0	
≤236.25	(reference)		(reference)			(reference)		(reference)		(reference)		(reference)	
Quartile 2	0.9173		0.9070			0.9954		0.8915		0.9205		0.9555	
236.25 to 472.5	(0.7730, 1.0886)		(0.7570, 1.0708)			(0.8247, 1.2015)		(0.7543, 1.0537)		(0.7743, 1.0537)		(0.7963, 1.1467)	
	<0.0001		<0.0001			<0.0001		<0.0001		<0.0001		<0.0001	
Quartile 3 472.5–708.05	0.9951		0.9003			1.1380		0.9612		0.9255		1.0824	
	(0.8417, 1.1765)		(0.7570, 1.0708)			(0.9545, 1.3569)		(0.8151, 1.1336)		(0.7792, 1.0992)		(0.9003, 1.3013)	
	<0.0001		<0.38			<0.0001		<0.0001		<0.0001		<0.0001	
Quartile 4 ≥708.05	1.3520		1.1380			1.5961		1.3487		1.1583		1.4418	
	(1.1482, 1.5920)		(0.9545, 1.3569)			(1.3127, 1.9406)		(1.1424, 1.5922)		(0.9688, 13849)		(1.1861, 1.7525)	
	<0.0001		<0.0001			<0.0001		<0.0001		<0.0001		<0.0001	
Per Quarter	0.9040		0.9778			1.3497		0.9716		1.0966		1.5164	
	(0.8021, 1.0189)		(0.8694, 1.0997)			(1.2014, 1.5163)		(0.8526, 1.1073)		(0.9618, 1.2502)		(1.3213, 1.2502)	
	<0.0001		<0.0001			<0.0001		<0.0001		<0.0001		<0.0001	
P Trend	<0.0001		<0.0001			<0.0001		<0.0001		<0.0001		<0.0001	
**Caffeine Intake**	**White Race**							**Nonwhite Race**					
	**Model 1**		**Model 2**			**Model 3**		**Model 1**		**Model 2**		**Model 3**	
Quartile 1	1.0		1.0			1.0		1.0		1.0		1.0	
≤236.25	(reference)		(reference)			(reference)		(reference)		(reference)		(reference)	
Quartile 2	0.8542		0.8285			0.9473		0.9448		0.9422		0.9801	
236.25 to 472.5	(0.7238, 1.0082)		(0.7001, 0.9804)			(0.7896, 1.1365)		(0.7943, 1.1238)		(0.7893, 1.1246)		(0.8115, 1.1839)	
	<0.0001		<0.0001			<0.0001		<0.0001		<0.0001		<0.0001	
Quartile 3	0.9509		0.8655			1.1136		0.9485		0.8845		1.0101	
472.5 to 708.05	(0.8123, 1.1133)		(0.7368, 1.0167)			(0.9333, 1.3287)		(0.7913, 1.1370)		(0.7352, 1.0641)		(0.8255, 1.2361)	
	<0.0001		<0.04			<0.0001		<0.0001		<0.0001		<0.0001	
Quartile 4 ≥708.05	1.2649		1.0496			1.5807		1.3716		1.1608		1.3181	
	(1.0838, 1.4763)		(0.8905, 1.2371)			(1.3131, 1.9027)		(1.1403, 1.6499)		(0.9586, 1.4056)		(1.0613, 1.6370)	
	<0.0001		<0.0001			<0.0001		<0.0001		<0.0001		<0.0001	
Per Quarter	0.8959		0.9563			1.3090		0.9671		1.0767		1.4731	
	(0.7948, 1.0099)		(0.8497, 1.0763)			(1.1638, 1.4722)		(0.8485, 1.1022)		(0.9438, 1.2283)		(1.2818, 1.6929)	
	<0.0001		<0.0001			<0.0001		<0.0001		<0.0001		<0.0001	
P Trend	<0.0001		<0.0001			<0.0001		<0.0001		<0.0001		<0.0001	
	**OR (95% CI), P value**												
**Caffeine Intake**	**2–5 years old**				**6–12 years old**					**13–19 years old**			
	**Model 1**	**Model 2**		**Model 3**	**Model 1**		**Model 2**		**Model 3**	**Model 1**	**Model 2**		**Model 3**
Quartile 1	1.0	1.0		1.0	1.0		1.0		1.0	1.0	1.0		1.0
≤236.25	(reference)	(reference)		(reference)	(reference)		(reference)		(reference)	(reference)	(reference)		(reference)
Quartile 2 236.25 to 472.5	0.9878	0.9965		1.0373	0.8667		0.8929		0.9229	0.8708	0.8811		0.9781
	(0.7851, 1.2429)	(0.7900, 1.2569)		(0.8054, 1.3360)	(0.7212, 1.0417)		(0.7411, 1.0758)		(0.7545, 1.1290)	(0.6944, 1.0922)	(0.7003, 1.1085)		(0.7614, 1.2565)
	<0.0001	<0.0001		<0.0001	<0.0001		<0.0001		<0.0001	<0.0001	<0.0001		<0.0001
Quartile 3 472.5 to 708.05	1.0715	1.0303		1.1704	0.9058		0.9389		1.0525	0.8539	0.8475		0.9476
	(0.8383, 1.3696)	(0.8029, 1.3222)		(0.8860, 1.5462)	(0.7573, 1.0834)		(0.7820, 1.1272)		(0.8610, 1.2866)	(0.6917, 1.0542)	(0.6838, 1.0504)		(0.7475, 1.2013)
	<0.0001	<0.0001		<0.0001	<0.0001		<0.0001		<0.0001	<0.0001	<0.0001		<0.0001
Quartile 4 ≥708.05	1.0207	1.0053		1.0500	1.2183		1.2874		1.4996	1.0980	1.1074		1.2321
	(0.6894, 1.5112)	(0.6767, 1.4935)		(0.6676, 1.6515)	(1.0054, 1.4764)		(1.0569, 1.5681)		(1.1997, 1.8745)	(0.9161, 1.360)	(0.9179, 1.3362)		(0.9924, 1597)
	<0.0001	<0.0001		<0.0001	<0.0001		<0.0001		<0.0001	<0.0001	<0.0001		<0.0001
Per Quarter	0.8965	0.9234		1.1645	0.9131		0.9266		1.1813	0.9609	1.0366		1.3411
	(0.7945, 1.0116)	(0.8199, 1.0400)		(1.0321, 1.3139)	(0.8081, 1.0317)		(0.8211, 1.0457)		(1.0438, 1.3369)	(0.8421, 1.0965)	(0.9079, 1.1835)		(1.1645, 1.5446)
	<0.0001	<0.0001		<0.0001	<0.0001		<0.0001		<0.0001	<0.0001	<0.0001		<0.0001
P Trend	<0.0001	<0.0001		<0.0001	<0.0001		<0.0001		<0.0001	<0.0001	<0.0001		<0.0001

Caffeine was adjusted for total energy intake with a residual model

Model 1: crude model.

Model 2: adjusted for gender, age, and race.

Model 3: adjusted for gender, age, race, the ratio of family income to poverty, total intake (from dietary and supplements), energy, protein, total sugar, total fat, cholesterol, vitamin B6, vitamin C, calcium, phosphorus, zinc, copper, sodium, phosphorus, and selenium.

In terms of race, in model 3, the ORs (95% CIs) of the prevalence of obesity for quartile 4 vs. quartile 1 were 1.5807 (1.3131, 1.9027) for the white race and 1.3181 (1.0613, 1.6370) <0.0001 for the nonwhite race. A per-quartile increase in caffeine intake was associated with a 16.38% increase and a 28.18% increased prevalence of obesity for white race and nonwhite race, respectively. The stratified analysis by race suggested that white race and nonwhite race were protective factors in the association between caffeine intake and the prevalence of obesity among children and adolescents.

Furthermore, age also affected the association between caffeine intake and the prevalence of obesity among children and adolescents. The ORs (95% CIs) of the prevalence of obesity for quartile 4 vs quartile 1 were 1.0500 (0.6676, 1.6515) for the age of 2–5, 1.4996 (1.1997, 1.8745) for the age of 6–12, and 1.2321 (0.9924, 1597) for the age of 13–19.

## Discussion

This study conducted a comprehensive analysis of the NHANES 2011–2020 March data to investigated the association between caffeine intake and the prevalence of obesity among children and adolescents. The findings indicated caffeine intake does not consistently correlate with obesity prevalence across different age groups.

The research obverse an inverse association betweent caffeine intake and obesity prevalence in certain quartiles of intake, suggesting a potential protective effect against obesity. This finding aligns with previous research highlighting caffeine’s role in enhancing metabolic rate and fat oxidation, which could contribute to a lower prevalence of obesity [4, 16]. However, this relationship was not uniform across all models, particularly after adjusting for various dietary and socioeconomic factors. This suggests that the association the caffeine and obesity is influenced by a complex interplay of multiple factors.

Notably, the subgroup in this research analysis indicated that the relationship between caffeine intake and obesity is modified by demographic variables like sex and race. These findings align with prior research indicating that genetic and environmental factors can alter metabolic responses to caffeine [5, 17–19]. Additionally, the nonlinear relationship we observed suggests that there is the potential for a threshold level of caffeine intake, beyond which its prevalence protective efforts against obesity diminish.

Despite the strength of our study, such as the robust NHANES dataset and the comprehensive analytical approach, the cross-sectional nature limits our ability to infer causality. Furthermore, the reliance on dietary recall data could introduce recall bias, and the estimation of caffeine intake needs to account for the variability in caffeine content across different food and beverage sources [2, 7, 13]. These limitations highlight the need for longitudinal studies to better understand the causal pathways linking caffeine intake with obesity prevalence.

Our study, utilizing the NHANES 2011–2020 data, revealed insightful findings about the association between caffeine intake and obesity among children and adolescents. We discovered that caffeine consumption does not consistently correlate with obesity across different demographics. In some intake quartiles, an inverse relationship between caffeine intake and obesity prevalence was noted, suggesting a possible protective role of caffeine. This aligns with prior research indicating caffeine’s metabolic benefits. However, the variability in this association after adjusting for dietary and socioeconomic factors suggests the influence of other confounding elements.

Importantly, our findings highlighted that demographic factors such as gender and race modify the caffeine-obesity relationship, supporting existing literature on the differential metabolic responses to caffeine. Moreover, the observed nonlinear relationship suggests a dose-response effect, indicating varying impacts of caffeine intake levels on obesity prevalence.

Despite the robustness of the NHANES dataset and the comprehensive nature of our analysis, the cross-sectional design limits causal inference. Additionally, the use of self-reported dietary data might introduce recall bias, and the variability in caffeine content in different foods and beverages must be considered. These limitations underscore the need for longitudinal studies for a deeper understanding of the causal pathways between caffeine intake and obesity.

## Conclusion

In our comprehensive analysis of the NHANES data from 2011 to 2020 March, we investigated the association between caffeine intake and obesity prevalence among children and adolescents. Our findings revealed a complex relationship in the age of 2–19. We observed that in some cases, caffeine intake appeared to have a protective effect against obesity, aligning with previous studies suggesting metabolic benefits of caffeine. However, this association varied significantly across different demographic groups, indicating that the impact of caffeine on obesity is not uniform. Meanwhile, subgroup analysis showed that factors like age, sex, and race significantly influenced the caffeine-obesity relationship, suggesting that metabolic responses to caffeine are diverse across populations. Furthermore, we noted a nonlinear relationship between caffeine intake and obesity prevalence, hinting at a dose-response effect.

## References

[1] Di Cesare M., Sorić M., Bovet P., Miranda J. J., Bhutta Z., Stevens G. A., et al. (2019). The epidemiological burden of obesity in childhood: a worldwide epidemic requiring urgent action. *BMC Medicine*, 17(1). doi: 10.1186/s12916-019-1449-8 31760948 PMC6876113

[2] Meiselman H. L. (2020). *Handbook of eating and drinking* *Volume* 1. Springer.

[3] Smith J. D., Fu E., & Kobayashi M. A. (2020). Prevention and Management of Childhood Obesity and Its Psychological and Health Comorbidities. *Annual Review of Clinical Psychology*, 16(1), 351–378. doi: 10.1146/annurev-clinpsy-100219-060201 32097572 PMC7259820

[4] Bustami M., Matalka K. Z., Mallah E., Abu-Qatouseh L., Abu Dayyih W., Hussein N., et al. (2021). The Prevalence of Overweight and Obesity Among Women in Jordan: A Risk Factor for Developing Chronic Diseases. *Journal of Multidisciplinary Healthcare*, *Volume* 14(1), 1533–1541. 10.2147/jmdh.s313172 34188480 PMC8235929

[5] Korekar G., Kumar A., & Ugale C. (2019). Occurrence, fate, persistence and remediation of caffeine: a review. *Environmental Science and Pollution Research*, 27(28), 34715–34733. 10.1007/s11356-019-06998-8 31811612

[6] Branum A. M., Rossen L. M., & Schoendorf K. C. (2014). Trends in Caffeine Intake Among US Children and Adolescents. *Pediatrics*, 133(3), 386–393. 10.1542/peds.2013-2877 24515508 PMC4736736

[7] Ye C., Xiao X., Sui H., Yang D., Yong L., & Song Y. (2023). Trends of caffeine intake from food and beverage among Chinese adults: 2004–2018. *Food and Chemical Toxicology*, 173(1), 113629. doi: 10.1016/j.fct.2023.113629 36682416

[8] Cornelis M. (2019). The Impact of Caffeine and Coffee on Human Health. *Nutrients*, 11(2), 416. doi: 10.3390/nu11020416 30781466 PMC6413001

[9] Barcelos R. P., Lima F. D., Carvalho N. R., Bresciani G., & Royes L. F. (2020). Caffeine effects on systemic metabolism, oxidative-inflammatory pathways, and exercise performance. *Nutrition Research*, 80(1), 1–17. doi: 10.1016/j.nutres.2020.05.005 32589582

[10] Abalo R. (2021). Coffee and Caffeine Consumption for Human Health. *Nutrients*, 13(9), 2918. doi: 10.3390/nu13092918 34578795 PMC8468147

[11] Bruci A., Tuccinardi D., Tozzi R., Balena A., Santucci S., Frontani R., et al. (2020). Very Low-Calorie Ketogenic Diet: A Safe and Effective Tool for Weight Loss in Patients with Obesity and Mild Kidney Failure. *Nutrients*, 12(2), 333. doi: 10.3390/nu12020333 32012661 PMC7071259

[12] Chao A. M., Quigley K. M., & Wadden T. A. (2021). Dietary interventions for obesity: clinical and mechanistic findings. *Journal of Clinical Investigation*, 131(1). doi: 10.1172/JCI140065 33393504 PMC7773341

[13] Hales C. M., Fryar C. D., Carroll M. D., Freedman D. S., & Ogden C. L. (2018). Trends in Obesity and Severe Obesity Prevalence in US Youth and Adults by Sex and Age, 2007–2008 to 2015–2016. *JAMA*, 319(16), 1723. doi: 10.1001/jama.2018.3060 29570750 PMC5876828

[14] McCormick D. P., Reyna L., & Reifsnider E. (2020). Calories, Caffeine and the Onset of Obesity in Young Children. *Academic Pediatrics*, 20(6), 801–808. doi: 10.1016/j.acap.2020.02.014 32081767 PMC7416448

[15] James J. E. (2020). Maternal caffeine consumption and pregnancy outcomes: a narrative review with implications for advice to mothers and mothers-to-be. *BMJ Evidence-Based Medicine*, 26(3), bmjebm-2020-111432. doi: 10.1136/bmjebm-2020-111432 32843532 PMC8165152

[16] Sirotkin A., & Kolesarova A. (2021). The anti-obesity and health-promoting effects of tea and coffee. *Physiological Research*, 70(2), 161–168. doi: 10.33549/physiolres.934674 33992045 PMC8820582

[17] Johnson C. E., Ryne Paulose-Ram, Ogden Cl, Carroll M., Kruszon-Moran D., Sm D., et al. (2013). National health and nutrition examination survey: analytic guidelines, 1999–2010. *PubMed*, 1(161), 1–24. 25090154

[18] Akomolafe S. F., Olasehinde T. A., Ogunsuyi O. B., Oyeleye S. I., & Oboh G. (2019). Caffeine improves sperm quality, modulates steroidogenic enzyme activities, restore testosterone levels and prevent oxidative damage in testicular and epididymal tissues of scopolamine-induced rat model of amnesia. *Journal of Pharmacy and Pharmacology*, 71(10), 1565–1575. doi: 10.1111/jphp.13142 31385305

[19] Guest N. S., VanDusseldorp T. A., Nelson M. T., Grgic J., Schoenfeld B. J., Jenkins N. D. M., et al. (2021). International society of sports nutrition position stand: caffeine and exercise performance. *Journal of the International Society of Sports Nutrition*, 18(1). doi: 10.1186/s12970-020-00383-4 33388079 PMC7777221

